# 

**Published:** 1897-02

**Authors:** 


FEBRUARY, 1897.

LIBRARY
Indexed
S. G. O.

### THE AMERICAN JOURNAL OF DENTAL SCIENCE.

PUBLISHED MONTHLY.

$2.50 PER ANNUM.

EDITED BY

F. J. S. GORGAS, M. D., D. D. S.
RICHARD GRADY, M. D., D. D. S.

Vol. 30. THIRD SERIES No. 10.

BALTIMORE:
SNOWDEN & COWMAN MFG. CO., 9 W. Fayette St.

LONDON:
TRUBNER & CO., 60 Paternoster Row.

Entered at the Post Office at Baltimore, Md., as Second-class Matter.

PAYABLE IN ADVANCE.

